# Supplementary material for: Remodeling hydrogen bond interactions results in relaxed specificity of Caspase-3
Source: Biosci Rep. 2021 Jan 29;41(1):BSR20203495. doi: 10.1042/BSR20203495 (PMC7846959; doi:10.1042/BSR20203495)
Supplement: Supplementary Figures S1-S4 [file BSR-2020-3495_supp.pdf]

Supplemental Figures

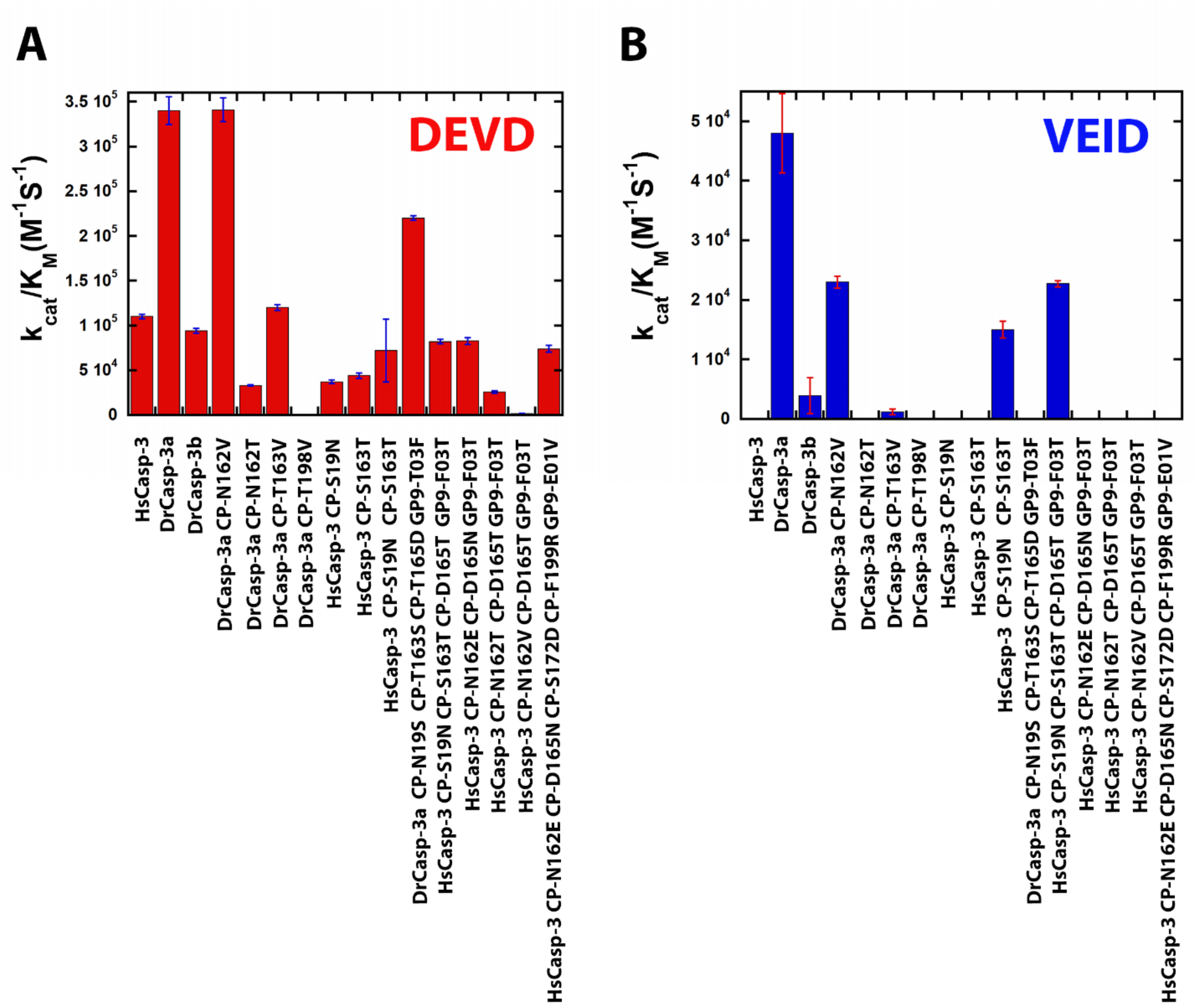

Supplemental Figure 1. Enzymatic activity of caspase mutants. Panels A,B. Enzyme specificity (k_{cat}/K_M) for wild-type and mutant caspases with DEVD (panel A) or VEID (panel B) as substrate. Data correspond to Table 2 in the main text.

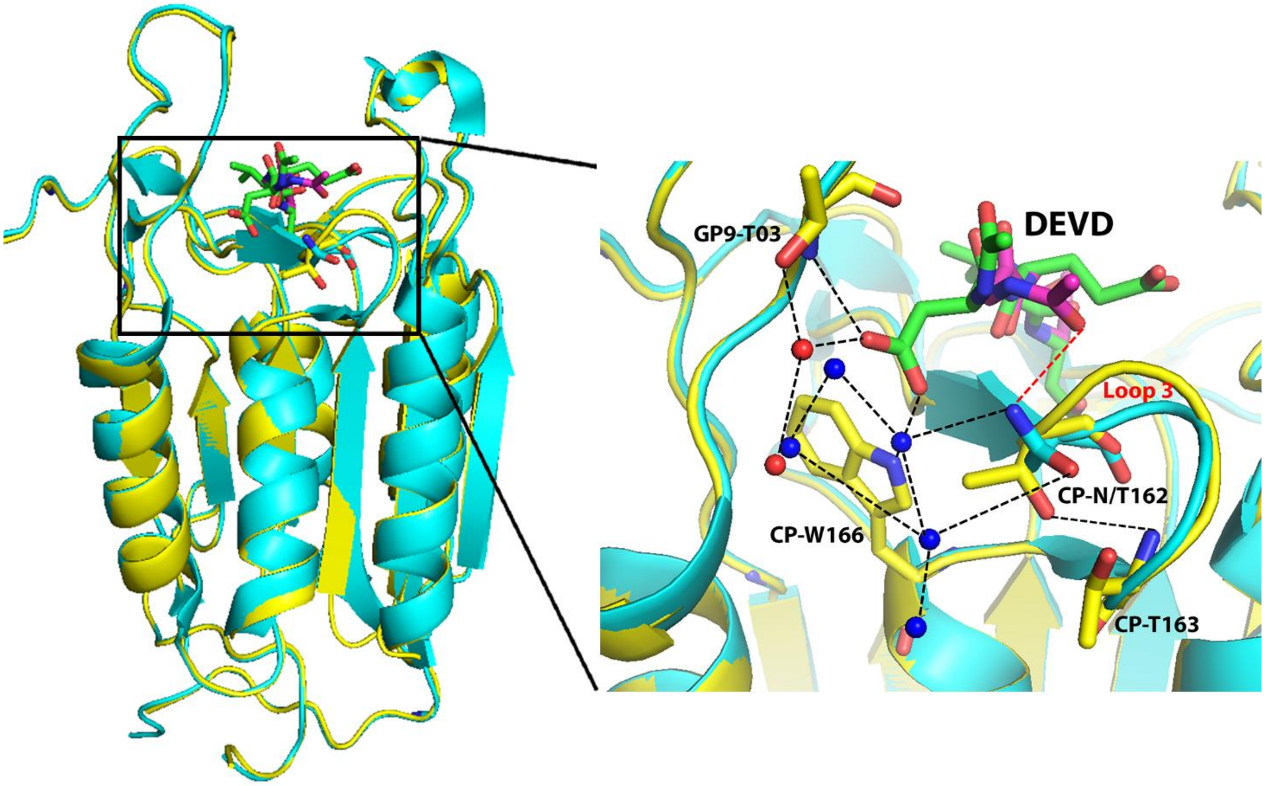

Supplemental Figure 2. Crystal structure of DrCasp-3a CP-N162T. Wild-type DrCasp-3a is shown in cyan, DrCasp-3a(CP-N162T) in yellow. The inhibitor, DEVD, is shown in magenta for wild-type DrCasp3a and in green for DrCasp3a(CP-N162T). Water molecules are shown in blue for wild-type DrCasp3a and in red for DrCasp3a (CP-N162T). The hydrogen bond between CP-N162 and the P4 aspartate residue of the substrate is shown in red. Loop 3 is labeled in red.

Supplemental Figure 3. Substrate specificity determined by substrate phage display. A-D. Substrate phage display for DrCasp-3b and mutants of HsCasp-3 and of DrCasp-3a. Sequence preferences are shown in the sequence logo inset for each panel. Results confirm DrCasp-3a(CP-N162T) and DrCasp3a(CP-N19S CP-T163S CP-T165D GP9-T03F) have high selection for DxxD, while HsCasp-3(CP-S19N CP-S163T CP-D165T GP9-F03T) have less selection for DxxD.

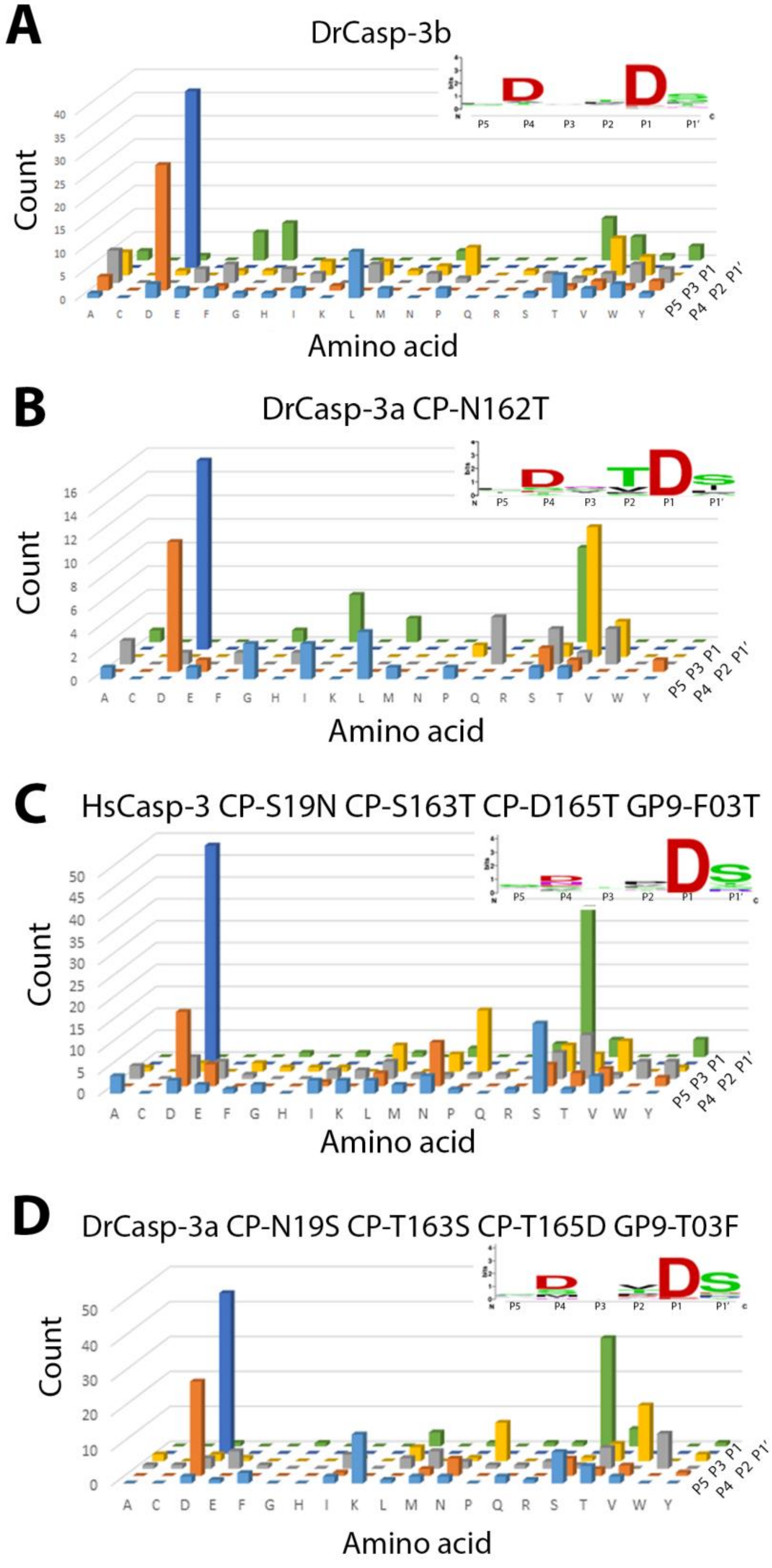

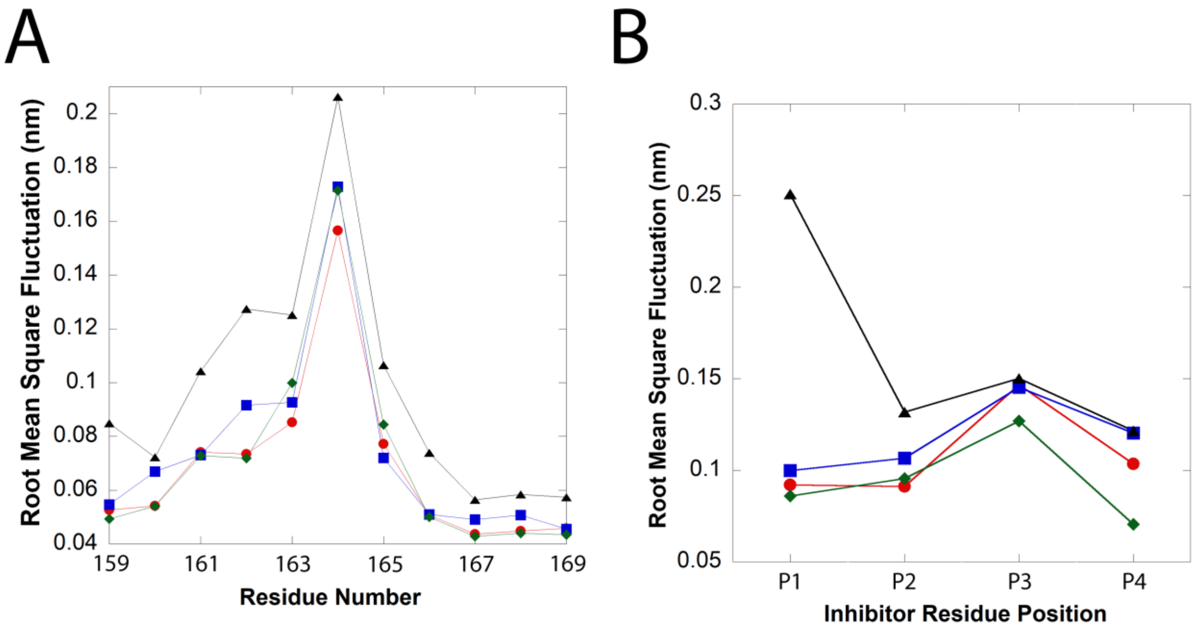

Supplemental Figure 4. The root-mean-square fluctuation (RMSF) for amino acids residues and inhibitor during MD simulation. A. RMSF for residues in DrCaspase-3a with DEVD binding (●) and VEID binding (■), compared to the RMSF for residues in DrCaspase-3a (CP-N19S CP-T163S) with DEVD binding(♦) and VEID binding (▲). B. The RMSF for DEVD while binding with DrCaspase-3a (●) and DrCaspase-3a (CP-N19S CP-T163S) (♦), compared to the RMSF for VEID binding with DrCaspase-3a (■) and DrCaspase-3a (CP-N19S CP-T163S) (▲).
